# Autophagic degradation of LOX-1 is involved in the maintenance of vascular integrity injured by oxLDL and protected by Berberine

**DOI:** 10.7150/ijbs.80958

**Published:** 2023-03-21

**Authors:** Yangmin Zheng, Bo Chen, Miaoqing Zhang, Yuanyuan Ma, Lulu Wang, Jingpu Zhang, Jiandong Jiang

**Affiliations:** NHC Key Laboratory of Biotechnology of Antibiotics, Beijing Key Laboratory of Antimicrobial Agents, Institute of Medicinal Biotechnology, Chinese Academy of Medical Sciences & Peking Union Medical College, Tian Tan Xi Li #1, Dongcheng District, Beijing 100050, China.

**Keywords:** autophagy, Berberine, cell co-culture, EMT, LOX-1, vascular injury, zebrafish

## Abstract

Damage to vascular endothelial cells (VECs) and vascular smooth muscle cells (VSMCs) caused by oxidized low-density lipoprotein (oxLDL) contributes to cardiovascular and cerebrovascular diseases. Protection effects of Berberine (BBR) on the cardiovascular system have been reported, however, the molecular mechanism of vascular protection is still unclear. In this study, we established two hyperlipidemia models in zebrafish and VEC-VSMC co-culture using high-cholesterol food (HCF) and oxLDL, respectively. We demonstrated that HCF doubled total cholesterol and total glyceride levels, and BBR decreased these indices in a concentration-dependent manner. Lipid staining and hematoxylin-eosin staining revealed that BBR inhibited oxLDL-induced VSMC bulge-like proliferation and migration toward VECs and prevented the HCF-induced trunk vascular obstruction in zebrafish. Immunoblot analysis, cell immunofluorescence, co-immunoprecipitation assays, and transmission electron microscopy showed that oxLDL/HCF increased lectin-like oxLDL receptor-1 (LOX-1) expression at least 5-fold and significantly inhibited autophagolysosome formation in the blood vessel cells and in zebrafish. These observations were associated with endothelial-to-mesenchymal transition (EMT) in VECs and triggered *VE-cadherin* ectopic expression in VSMCs, and they were responsible for aberrant VSMC migration and vascular occlusion. However, BBR, by promoting autolysosome formation and degradation of LOX-1, reversed the above events and maintained intracellular homeostasis of vessel cells and vascular integrity. In conclusion, regulation of autophagy may be an effective approach to treating oxLDL-induced cardiovascular diseases by reducing LOX-1 protein level. BBR can protect blood vessels by adjusting the oxLDL-LOX-1-EMT-autophagy axis. This study is a step toward the development of new applications of BBR.

## Introduction

Hyperlipidemia-related cardiovascular diseases are involved in complicated vascular pathogenesis with a chronic process 1. Clinical studies suggest that lipid metabolism disorder and high-cholesterol food (HCF) may be major risk factors for the development of acquired cardiovascular diseases 2-4. Cholesterol depositions are usually found in atherosclerotic plaques in patients with coronary heart disease 4, 5. Lectin-like oxidized low-density lipoprotein receptor-1 (LOX-1) can mediate oxidized low-density lipoprotein (oxLDL) uptake in vascular endothelial cells (VECs) 6, and contributes to the formation of atherosclerosis 7. High expression of LOX-1 protein was found in patients with atherosclerosis and in VECs and vascular smooth muscle cells (VSMCs) derived from animal models 8. Further studies in VSMCs or VECs have shown that oxLDL could increase levels of LOX-1 protein and simultaneously block the autophagic processes 9. Autophagy, one of mechanisms underlying cell homeostasis, is a process in which intracellular abnormal or excess proteins and damaged organelles are degraded 10, 11. VECs, VSMCs and macrophages are the most important cell types involved in the formation of atherosclerosis 12. Studies have shown that activation of autophagy inhibits inflammation and apoptosis of VECs and VSMCs, promotes cholesterol efflux 13, and reduces LOX-1 protein levels 14. Downregulation of autophagy might mediate VSMC-derived foam cell formation in advanced atherosclerosis 15 and increase LOX-1 protein levels 14. These observations indicate that maintenance of normal autophagic activity may be a treatment strategy for atherosclerosis. However, the above results were separately derived from different laboratories and from different periods; thus, a direct relationship among hyperlipidemia, oxLDL/LOX-1, autophagy, and vascular injury need to be confirmed by a systematically integrated study.

Berberine (BBR) is the main active component of *Coptis chinensis*, *Berberis*, and etc, and is used as a traditional Chinese medicine in clinical treatment of diarrhea and bacterial dysentery 16. Recently, BBR has been found to have new potential therapeutic effects on metabolic diseases, such as diabetes 17, 18, hyperlipidemia 19, 20 and cardiovascular diseases 4, 21. Studies of molecular mechanism have shown that BBR activates the extracellular-regulated protein kinase pathway, increases the expression of LDL receptor on the surface of liver cells 19, and attenuates cholesterol accumulation in macrophage foam cells by suppressing AP-1 activity and activating the Nrf2/HO-1 pathway 22. Also, BBR could suppress expression of LOX-1 in HUVECs and monocyte/macrophages 23, 24. The stimulation of autophagy by BBR was reported in macrophages and liver 25-28 but the association with blood vascular protection has not been verified. BBR, a potential drug for treatment of cardiovascular diseases, has attracted attention of the medicinal field 29, 30, but the mechanisms underlying its wide range of effects are still elusive. In the present study, we aimed to illustrate whether and how the inhibitory effects of BBR on high-fat-induced cardiovascular diseases are directly correlated to its stimulatory effects on autophagy. We established HCF/oxLDL-induced vascular injury models using zebrafish and co-culture of HUVECs and HVSMC, respectively. Mechanism of BBR regulating intracellular homeostasis of vascular cells in response to HCF/oxLDL induction was investigated. We found that BBR promoted autophagolysosome formation and regulated the oxLDL-LOX-1-endothelial-to-mesenchymal transition (EMT)-autophagy axis in blood vessel cells *in vitro* and *in vivo*. This study provides evidence that autophagy is important in the maintenance of intracellular homeostasis of vascular cells by degrading LOX-1 protein.

## Results

### BBR alleviates lipid accumulation in zebrafish

**Figure 1:**
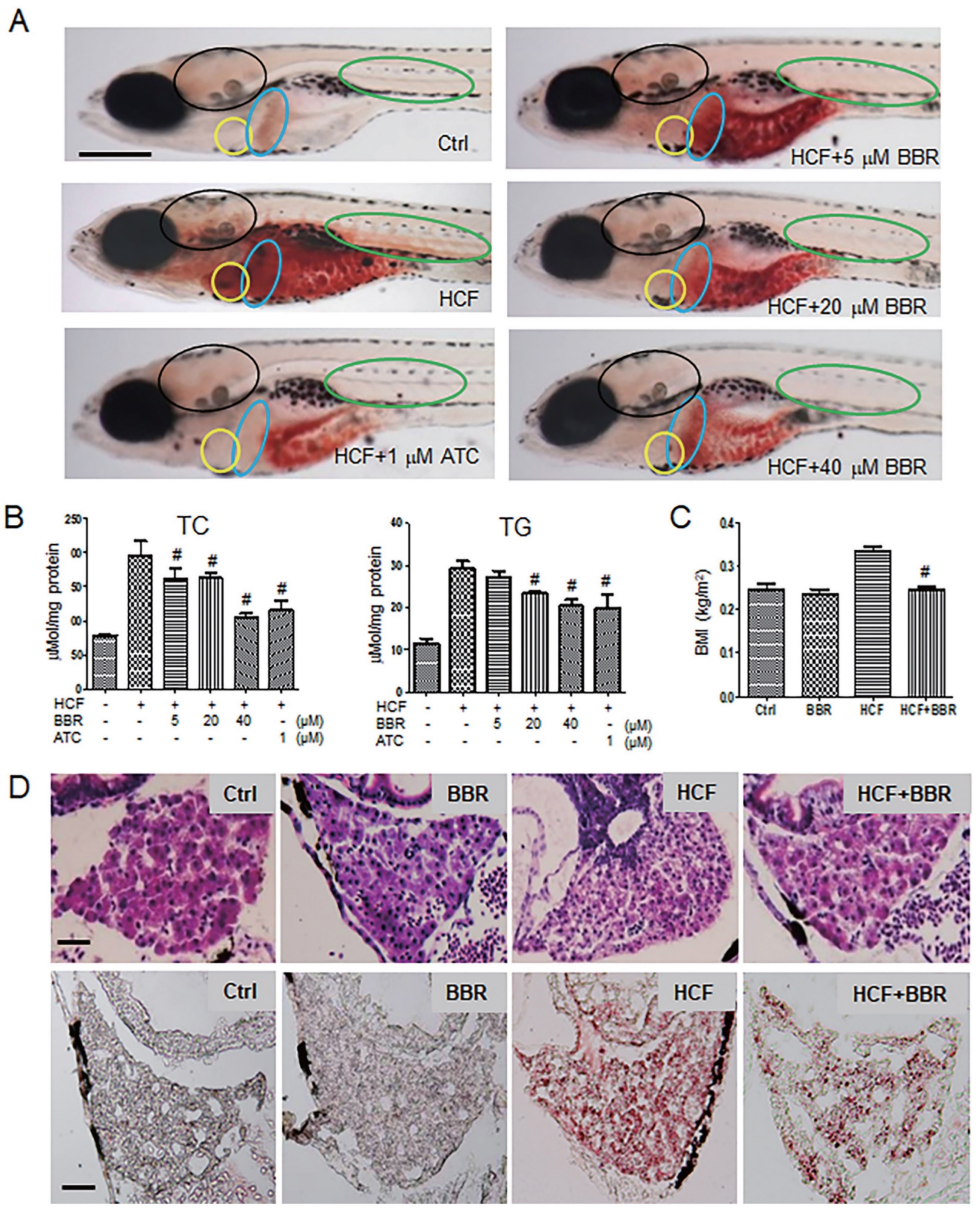
** Effects of BBR on the HCF-induced disorders of lipid metabolism in zebrafish larvae. A** Attenuation of the HCF-induced lipid accumulation by BBR, as indicated by whole-body ORO staining (n = 20). Colored ovals indicate the following tissues: heart (yellow oval circle), liver (blue oval circle), brain (black oval circle), and trunk vessels (green oval circle). The scale bar represents 200 μm. **B** Total cholesterol (TC) and total triglycerides (TG) were reduced at the administered concentration of BBR. Samples were collected from 20 zebrafish and tested in triplicate. **C** Abnormal BMI was corrected by BBR treatment. The BMI was calculated as the ratio of body weight (kg) to body length squared (m^2^) (n = 10). **D** BBR-related reduction of high-cholesterol-induced lipid accumulation in liver in both paraffin sections with hematoxylin-eosin staining (HE staining, upper panel) and frozen sections with ORO staining (lower panel) (n = 10). The scale bars represent 15 μm. BBR concentration was 20 μM in (**C**) and (**D**). #*P* < 0.05 *vs* HCF. BBR: berberine; HCF: high-cholesterol food; and ORO: Oil Red O; ATC: Atorvastatin (positive control).

To investigate the protective effects of BBR on the hyperlipidemic cardiovascular vessels and the underlying molecular mechanism, we first established a hyperlipidemia model in zebrafish following a feeding flowchart (Fig. S1A). A LC-MS/MS assay indicated that BBR was indeed absorbed by the zebrafish body (Table S1). Oil red-O staining revealed that zebrafish that were fed with HCF showed severe lipid accumulation in the heart, brain, liver, abdomen, and trunk blood vessels compared with the control group. BBR strongly reduced or eliminated lipid accumulation in a concentration-dependent manner (Fig. 1A). Biochemical assays showed that BBR also reduced the total cholesterol and triglyceride levels (Fig. 1B), and significantly lowered the body mass index (BMI) of zebrafish fed HCF (Fig. 1C). Moreover, observations from Hematoxylin-eosin (HE) staining and frozen sections of liver tissues showed that HCF-induced fatty vesicle accumulation was attenuated by BBR treatment (Fig. 1D).

### BBR reverses the vessel occlusion aggravated by human LOX-1 in the zebrafish hyperlipidemia model

**Figure 2:**
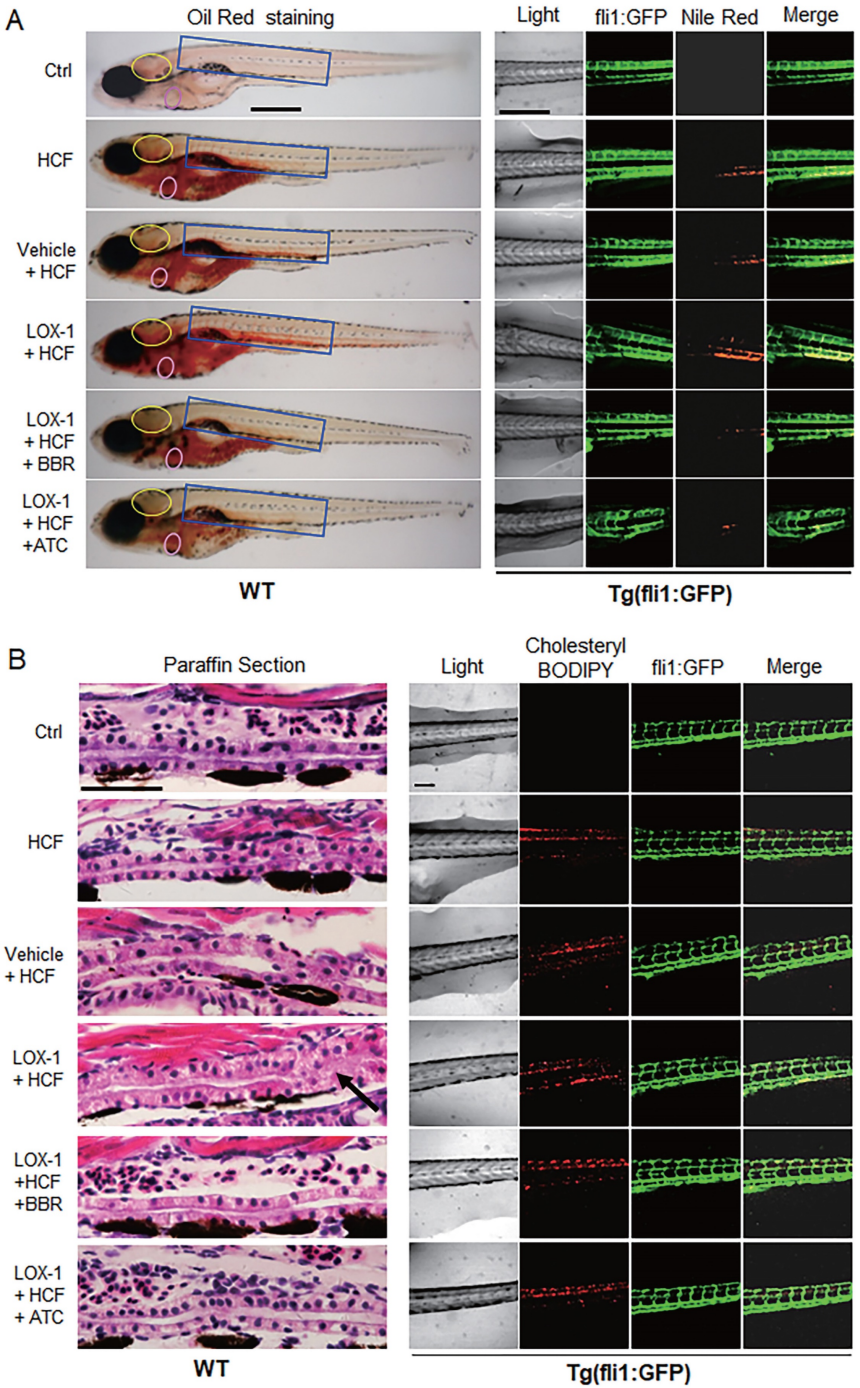
** BBR attenuated vascular lipid accumulation and vascular occlusion aggravated by LOX-1 in the HCF-treated zebrafish. A** Lipid accumulation in blood vasculature and heart was observed by whole-body ORO staining and Nile Red staining (n = 20). ORO-stained blood vessels are observed at the following body parts: the brainstem (yellow oval ring), the heart (pink oval ring), and the trunk (blue rectangle) (left panel). In the Nile Red staining of the Tg(fli1:GFP) larvae, the green fluorescence represents blood vessels, and the red fluorescence indicates lipid deposition (right panel). The bars indicate 300 μm. **B** Visible pathological changes of blood vessel structure in zebrafish using HE staining of paraffin sections (left panel). The black arrow points at a part of the blocked vessel. BBR (20 μM) and ATC (1 μM) were respectively given to the HCF plus *LOX-1* group. Cholesterol deposition (marked by cholesteryl-BODIPY) in blood vessel walls was observed in transgenic zebrafish Tg(fli1:GFP) using a laser scanning confocal microscope (n = 10) (right panel). The bars indicate 100 μm.

Considering that LOX-1 protein is a receptor of oxLDL in VECs and VSMCs, we examined whether expression of human LOX-1 protein played a role in HCF-induced lipid accumulation and vascular occlusion in zebrafish. A zebrafish *Fli1* promoter fragment was cloned to construct an expression vector of human *LOX-1* gene that could be expressed specifically in zebrafish VECs. The expression of human LOX-1 was sustained until at least 15 days post-fertilization (dpf) using western blotting test (Fig. S1B), meeting the experimental requirements. Oil Red O staining and Nile Red staining, compared with the HCF alone group, showed that overexpression of human LOX-1 led to more serious lipid accumulation and that BBR significantly reduced lipid levels in the blood vessels of the brain and trunk (Fig. 2A). Furthermore, using the HCF supplement cholesteryl-BODIPY to identify the deposition of cholesterol in the body of Tg(fli:gfp) fish, we found that the expression of human LOX-1 protein aggravated the deposition of cholesterol in the trunk vessels (Fig. 2B, right part). HE staining analysis furthermore showed that the upregulation of LOX-1 protein caused a disordered vascular structure and impeded blood flow in zebrafish trunk vessels (Fig. 2B, left part)). BBR significantly attenuated the luminal blockage (Fig. 2B, left part). Further, the anti-atherosclerotic effect of BBR was verified in a mouse model using ApoE^(-/-)^ mice fed a high fat diet (HFD). Oil Red O staining revealed that the mice in the HFD group developed more arterial plaques than those in the negative control group, and BBR treatment significantly reduced the arterial plaque formation and lipid deposition (Fig. S2).

### BBR inhibits oxLDL-induced proliferation of VSMCs and endothelial-to-mesenchymal transition of VECs

**Figure 3:**
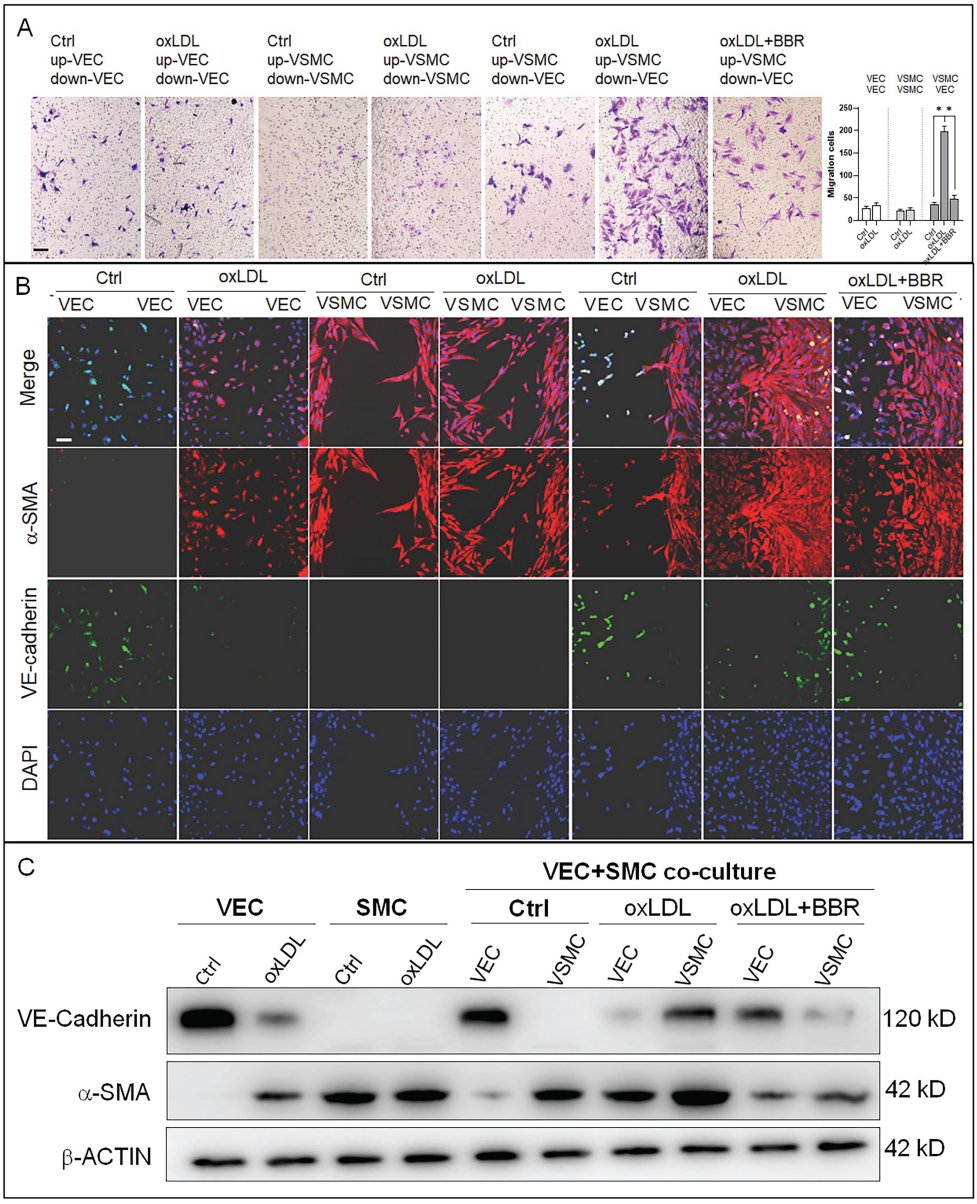
** BBR-related inhibition of oxLDL-induced bulge-like proliferation of VSMCs and EMT of VECs. A** VSMC migration ability was measured by Transwell assays (scale bar, 200 µm). **B** Cell immunofluorescence assay confirming the VSMC migration and ectopic expression of VEC and VSMC marker proteins. The antibody against α-SMA protein is labeled with red fluorescence, and the anti-VE-cadherin antibody is labeled with green fluorescence (scale bar, 50 μm). **C** Western blot results show the levels of VE-cadherin and α-SMA proteins affected by the different treatments. The western blot results from the co-culture condition were semi-quantitatively analyzed, and the data are presented in Fig. **S3C** (n = 5). The used concentrations were 1 μM for BBR and 100 μg/mL for oxLDL. VEC: human umbilical vein endothelial cells; VSMC: human vascular smooth muscle cells.

To research the molecular mechanisms underlying the effects of oxLDL and BBR in blood cells, we established a co-cultivation model of VECs and VSMCs to simulate the basic vascular structure (Fig. S3A). Using this double-cell model, we found that oxLDL induced VSMC proliferation and migration with bulges toward the VEC side compared the co-culture and monoculture groups without oxLDL. In response to BBR administration to the two-cell co-cultures exposed to oxLDL, VSMC proliferation and migration were significantly reduced (Fig. S3B). Then, the VSMC migration was confirmed using a transwell assay. Under the monoculture of VEC or VSMC, oxLDL did not obviously affect cell migration compared to the control groups without oxLDL. Under VEC-VSMC co-cultures, oxLDL greatly stimulated VSMC migration to VEC and BBR notably inhibited the migration compared with the control group (Fig. 3A). In cell immunofluorescence experiment, the VSMC marker alpha-smooth muscle actin (α-SMA) and the VEC marker VE-cadherin were used to visualize the interaction and transition of the marker proteins between VECs and VSMCs. The results showed that VE-cadherin protein was greatly reduced, while α-SMA protein was ectopically detectable in the VECs of monoculture plus oxLDL, and obviously aggravated in the VECs of VEC-VSMC co-culture plus oxLDL, implying endothelial-to-mesenchymal transition (EMT) occurrence in VECs in response to oxLDL-induction. Additionally, under VEC-VSMC co-culture plus oxLDL, VSMCs ectopically expressed VE-cadherin, and proliferated and migrated toward VECs compared to the VSMC monocultures with or without oxLDL induction. The abnormal proliferation of VSMC and the interchanged expression of the marker proteins between VSMCs and VECs were significantly attenuated or eliminated by BBR (Fig. 3B). These results were confirmed by western blot assays (Fig. 3C and Fig. S3C). OxLDL significantly inhibited VE-cadherin expression but triggered ectopic α-SMA expression in VECs in the VEC monocultures and in VEC-VSMC co-cultures; in VSMCs, oxLDL strongly triggered ectopic expression of VE-cadherin and enhanced α-SMA level in the VEC-VSMC co-cultures. BBR greatly reversed these aberrant changes to near the control groups. These results show BBR protection of blood vessels via suppressing the ectopic expression of the genes induced by oxLDL. The interaction between VECs and VSMCs is a non-negligible factor.

### LOX-1 mediates oxLDL-induced “sprouted” proliferation of VSMCs and EMT of VECs

**Figure 4:**
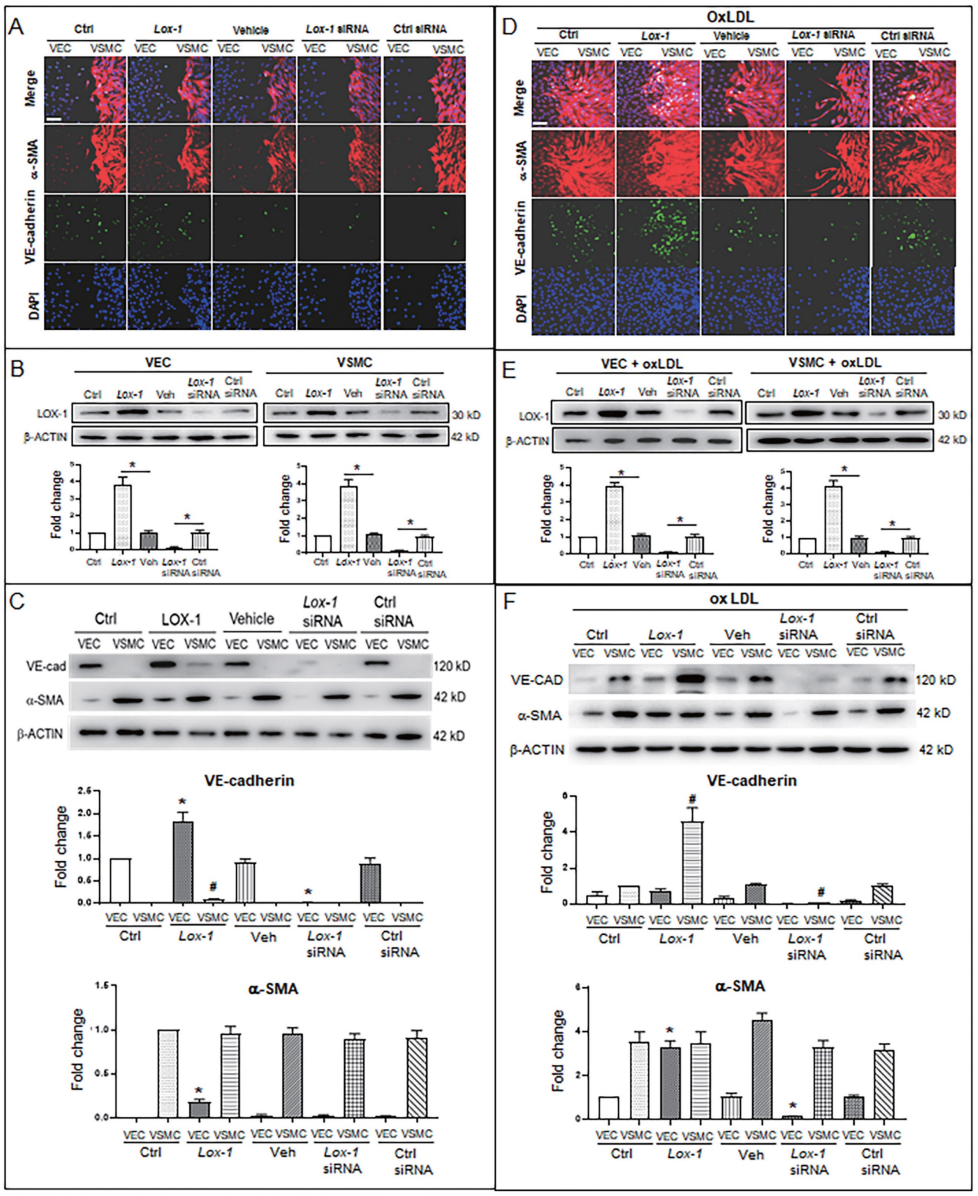
** LOX-1 promotes proliferation of VSMCs and ectopic expression of protein markers in VECs and VSMCs.** LOX-1 protein was up- or downregulated by transfection of pLOX1-IRES2-EGFP or *lox1*-siRNA, respectively, in both VECs and VSMCs. **A, D** Cell immunofluorescence experiments show the extent of proliferation and migration of VECs and VSMCs of the double-cell co-culture in the absence (**A**) or presence (**D**) of oxLDL. Red color indicates α-SMA, and green color indicates VE-cadherin. The bars indicate 60 μm in (**A**) and (**D**). **B, E** Western blots show levels of LOX-1 protein after transfection with the LOX-1 overexpression vector and siRNA, respectively in both VSMC and VEC in the absence (**B**) or presence (**E**) of oxLDL (n = 5). *P < 0.05*.* **C, F** Western blots indicate shifting protein levels of VE-cadherin and α-SMA affected by *lox-1* overexpression or siRNA knockdown in the absence (**C**) or presence (**F**) of oxLDL (n = 5). *P < 0.05 *vs* VEC ctrl, #*P* < 0.05 *vs* VSMC ctrl. The oxLDL concentration was 100 μg/mL.

Further, considering LOX-1 being an important receptor of oxLDL, we used a *lox1* overexpression vector and *lox1*-siRNA to check if the level of LOX-1 was implicated in oxLDL-induced abnormal proliferation of VSMCs. When oxLDL was not administered, LOX-1 overexpression showed a moderate effect on bulged cell growth of VSMCs; LOX-1 downregulation inhibited the bulged growth of VSMCs compared with the groups in the double-cell co-cultures (Fig. 4A). Western blot analysis shows that LOX-1 levels (Fig. 4B) are positively related with the degree of VSMC proliferation. In the double cell co-culture without oxLDL, VE-cadherin protein was produced in VECs of control groups, but not in the corresponding VSMC groups; however, LOX-1 overexpression moderately raised VE-cadherin protein level in VECs and ectopically initiated VE-cadherin production in VSMCs; suppression of LOX-1 expression by *lox-1* siRNA inhibited VE-cadherin expression in both VECs and VSMCs. Both LOX-1 overexpression and LOX-1 knockdown did not affect the level of α-SMA protein in VSMCs, but the LOX-1 level was positively correlated with the production of α-SMA protein in VECs (Fig. 4C). When oxLDL was added to the double-cell co-culture medium, the four VSMC groups (three controls and LOX-1 overexpression) presented significant “bulge” growth toward the VEC region, the LOX-1 overexpression group showed the biggest degree of VSMC “bulge” proliferation and migration, which did not occur among cells with downregulation of LOX-1 (Fig. 4D). Western blot analysis showed that the levels of LOX-1 protein in both VECs and VSMCs (Fig. 4E) were positively correlated with the intensity of bulge-like cell proliferation and with the VE-cadherin level in VSMCs in the different groups (Fig. 4D). Further, the altered protein levels of VE-cadherin and α-SMA were confirmed by western blot analysis (Fig. 4F). Under oxLDL treatment, the level of VE-cadherin protein was notably elevated in VSMCs, particularly in the LOX-1 overexpression group; on the contrary, the levels of VE-cadherin protein were significantly reduced in VECs (Fig. 4F), compared with the non-oxLDL groups (Fig. 4C). On the other hands, the level of α-SMA protein was not affected in the five groups of VSMCs; however, it was unexpectedly elevated in the VEC groups including the control groups and the LOX-1 overexpression group, and decreased greatly only in the LOX-1 knockdown VECs (Fig. 4F). These results suggests that LOX-1 mediates the oxLDL-induced VSMC “bulge”-like proliferation and migration and induced both ectopic expressions of VE-cadherin in VSMCs and α-SMA in VECs, hinting at EMT occurrence and a role of the VE-cadherin protein in VSMC proliferation and migration.

### BBR suppresses the ectopic expression of *VE-cadherin* and *α-SMA* genes and induces degradation of LOX-1 protein by promoting of autolysosome formation in VECs and VSMCs

**Figure 5:**
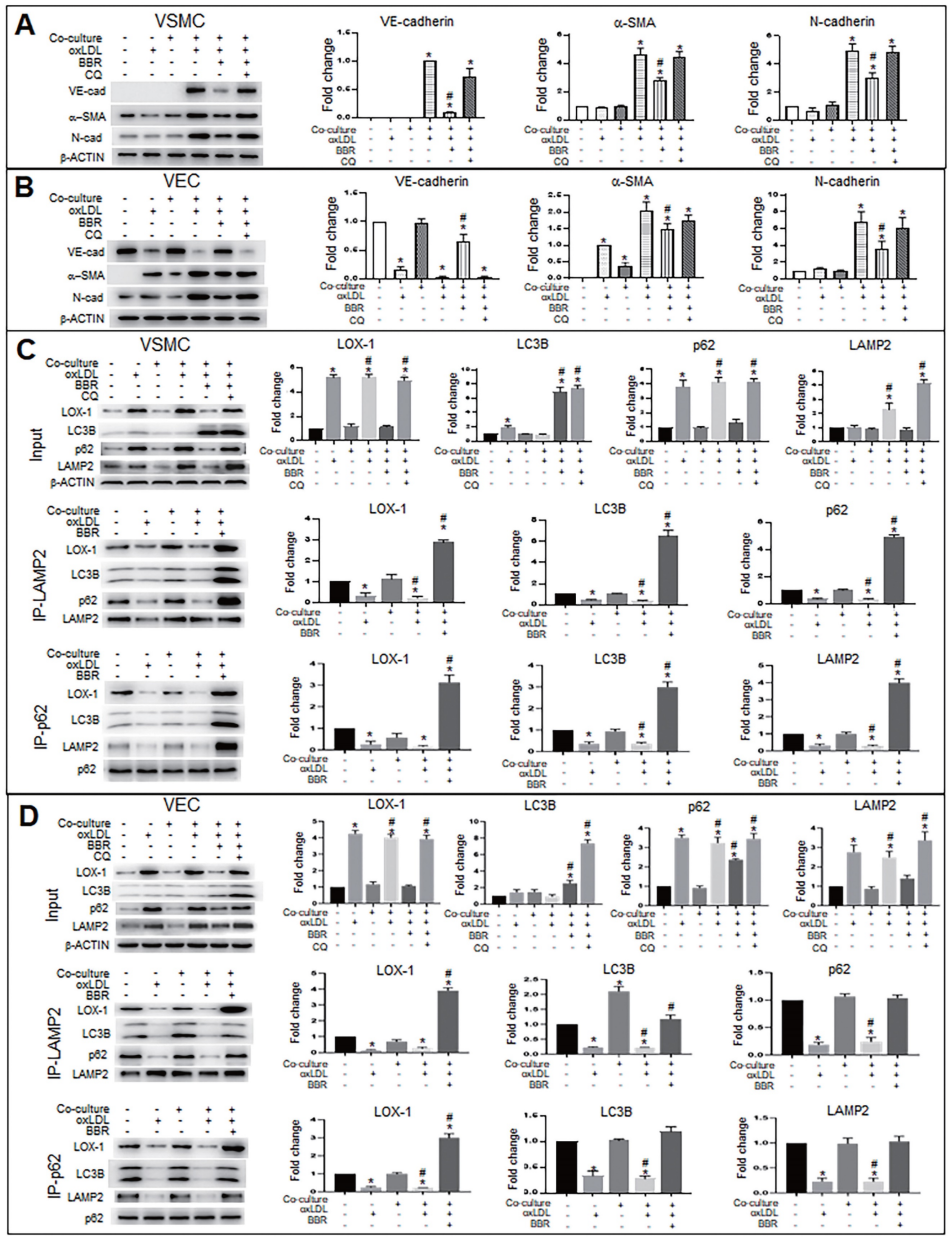
** BBR inhibits EMT and decreases LOX-1 via promotion of autolysosomal digestion in VSMCs and VECs. A, B** Western blot analysis shows that the autophagy inhibitor CQ blocked BBR's anti-EMT role in VSMCs (**A**) and VECs (**B**). *P < 0.05 *vs* monoculture ctrl of VSMC (**A**) or VEC (**B**), #*P* < 0.05 *vs* VSMC (**A**) or *vs* VEC (**B**) under VEC-VSMC co-culture plus oxLDL. **C, D** Co-immunoprecipitation analysis with anti-p62 and anti-LAMP2 antibodies shows the interaction between the autophagy proteins p62, LC3B, and LAMP2 and LOX1 promoted by BBR in VSMCs (**C**) and VECs (**D**) under the condition of VEC-VSMC co-culture plus oxLDL. All western blot data were from independent tests (n = 4 or 5). *P < 0.05 *vs* monoculture ctrl of VSMCs (**C**) or VECs (**D**); #*P* < 0.05 *vs* co-culture ctrl of VSMCs (**C**) or VECs (**D**).

To explore the molecular mechanism of autophagy on BBR suppression for both the EMT and the gene ectopic expression, we used the autophagy inhibitor chloroquine (CQ) to examine levels of EMT marker proteins VE-cadherin, α-SMA and N-cadherin under conditions of VEC-VSMC co-culture plus oxLDL and with or without BBR. In the VSMCs (Fig. 5A), the VEC marker VE-cadherin appeared in the three VSMC groups of the co-culture plus oxLDL; of the three, the VE-cadherin level obviously decreased in BBR treatment group and raised in the BBR plus CQ group, compared with the co-culture without oxLDL group. Both α-SMA and N-cadherin are markers of mesenchymal cells and also increased in the three co-culture plus oxLDL groups, but downregulated in the BBR-added group. In the three groups of VECs plus oxLDL (Fig. 5B), VE-cadherin protein was significantly reduced compared with the two groups without oxLDL, but the VE-cadherin level was significantly recovered in the BBR treatment group and lowered again in the BBR plus CQ group. Additionally, BBR reduced the levels of α-SMA and N-cadherin proteins and CQ blocked the BBR effects. Thus, we infer that oxLDL induced both EMT likelihood in VECs and the ectopic expression of *VE-cadherin* gene in VSMCs, which were probably reversed by the pro-autophagy activity of BBR.

Considering the LOX-1 role in the ectopic expression of VE-cadherin and α-SMA, we next determined whether autophagy mediated the BBR effect on degradation of excessive LOX-1 protein. *Lox-1* transcript levels were first determined using qRT-PCR (Fig. S4), which showed that oxLDL significantly increased *lox-1* mRNA levels in both VSMCs and VECs and BBR did not affect *lox-1* mRNA levels under oxLDL exposure. Co-immunoprecipitation (Co-IP) experiments (Fig. 5C, D) showed that under both the cell monoculture and double-cell co-culture, the protein levels of LOX-1, autophagic substrate receptor p62/sequestosome 1 (p62) and lysosomal marker lysosomal associated membrane protein 2 (LAMP2), but not LC3B, were notably higher in oxLDL-treated groups than in those without oxLDL, indicating blockade of autophagic degradation; BBR treatment significantly reduced LOX-1, p62 and LAMP2, but increased the level of the autophagy marker LC3B-II, reflecting BBR-related stimulation of autophagic activity and lysosomal degradation activity. However, when CQ was added to the co-culture media, which reduces autolysosome acidity, the effects of BBR were blocked. Co-IP results further verified that oxLDL inhibited conjunction among the proteins p62, LOX-1, LC3B and LAMP2, meaning that both autophagolysosome formation and LOX-1 transportation to the autophagolysosomes were blocked. BBR restored the combination of the four proteins, and hence autophagolysosomes were formed and LOX-1 was transferred into the autophagolysosomes. These results suggest that one of the BBR functions is promoting autophagolysosome formation which causes degradation of LOX-1 protein in both VSMCs and VECs.

**Figure 6:**
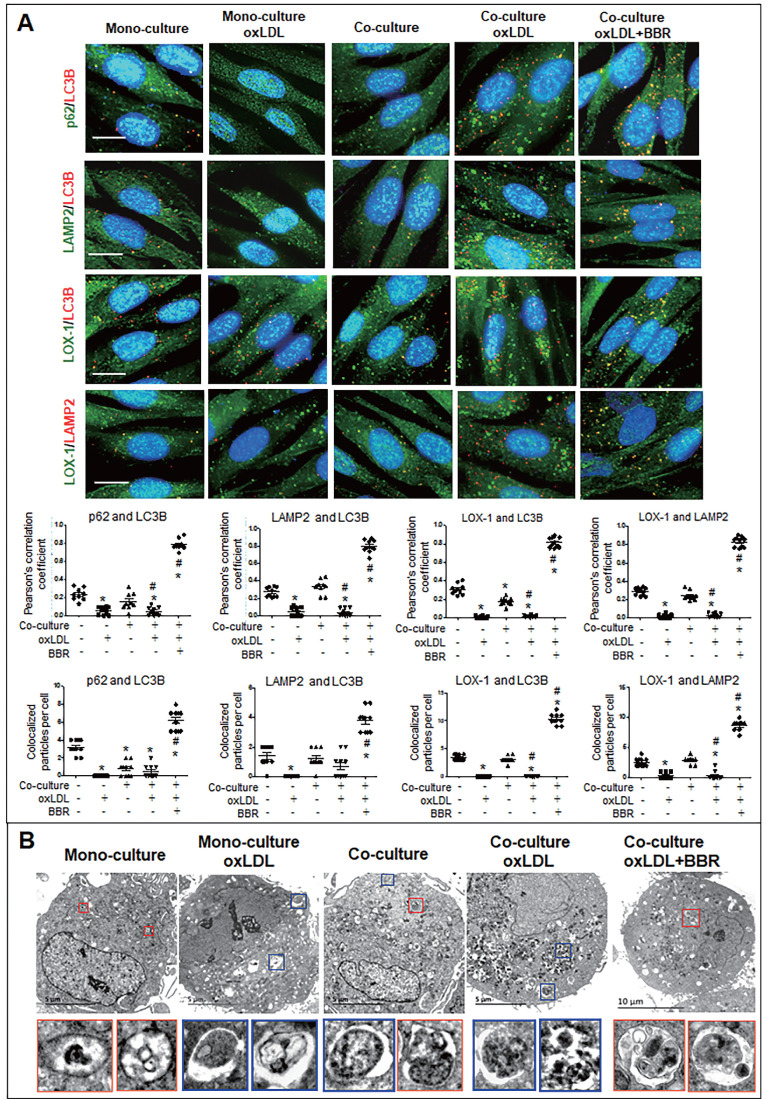
** BBR induction of autolysosome formation in VSMC. A** Cell immunofluorescence analysis showing the subcellular co-localization of p62-LC3B, LAMP2-LC3B, LOX-1-LC3B and LOX-1-LAMP2 with their Pearson correlation coefficients and the particle counts (n = 10). The detailed figures of the colocalization particles are shown in Fig. **S5A** and **B**. The bars indicate 15 μm. **B** showing Autophagosomes and autolysosomes in VSMCs under monoculture and co-culture conditions as observed by transmission electron microscopy, and the particle numbers of autophagosomes and autolysosomes are shown in Fig. **S5C** (n = 20). The BBR concentration was 1 μM, and the oxLDL was 100 μg/mL. Blue boxes indicate representative autophagosomes and red boxes indicate representative autolysosomes or amphisomes, and these structures are enlarged below. The aggregated autophagosomes are shown in the right blue box in the group of co-culture plus oxLDL.

**Figure 7:**
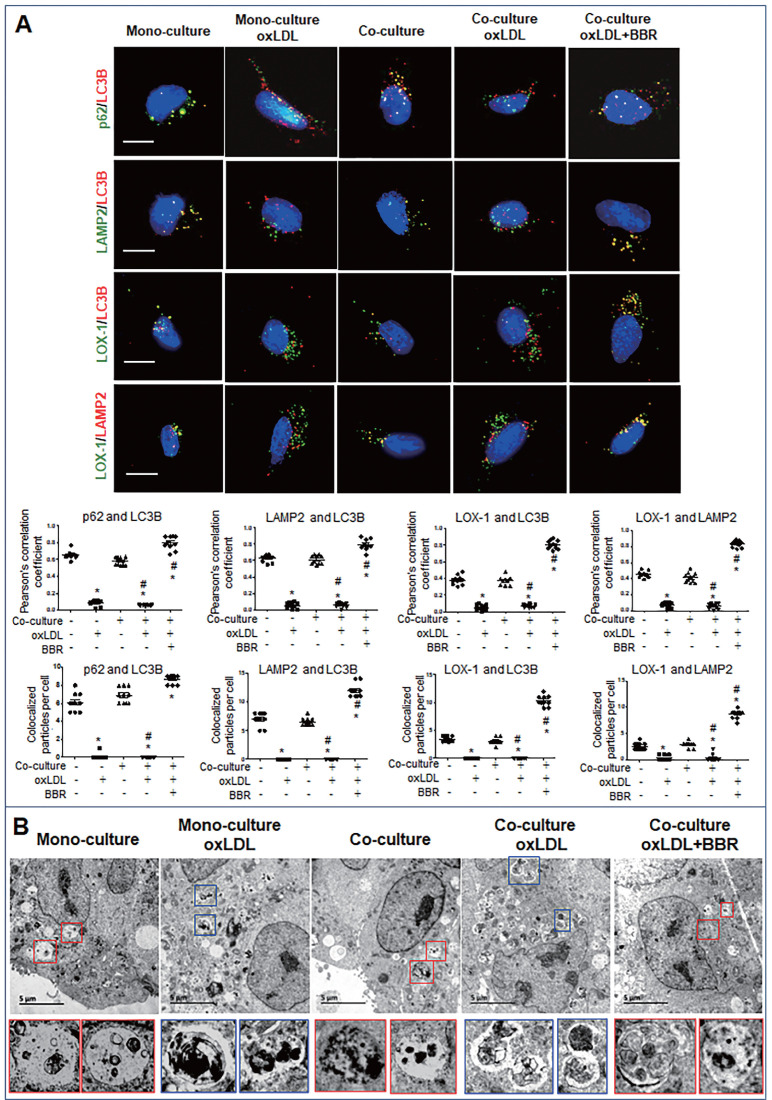
** BBR induction of autolysosome formation in VECs. A** Cell immunofluorescence analysis showing the subcellular co-localization of p62-LC3B, LAMP2-LC3B, LOX-1-LC3B and LOX-1-LAMP2 with their Pearson correlation coefficients and particle numbers (n=10), respectively. The detailed figures of the colocalized particles are shown in Fig. **S6A** and **B**. The bars indicate 15 μm. **B** Autophagosomes and autolysosomes in VECs were observed by transmission electron microscopy, and the particle numbers of autophagosomes and autolysosomes are shown in Fig. **S6C** (n = 20). Blue boxes indicate representative autophagosomes and red boxes indicate representative autolysosomes or amphisomes, and these structures are amplified below. The BBR concentration was 1 μM, and the oxLDL was 100 μg/mL. The bars indicate 5 μm.

Furthermore, cell immunofluorescence experiments indicated that under the VSMC-VEC co-culture plus oxLDL condition, LC3B, p62, and LAMP2 showed no co-localization in VSMCs and VECs compared to the groups without oxLDL, confirming inhibition of oxLDL on autophagosome formation. When BBR was added, four kinds of co-localized particles including LC3B-p62, LC3B-LAMP2, LOX-1-LC3B and LOX-1-LAMP2 were observed and supported by high Pearson's correlation coefficients (Figs. 6A, 7A; Figs. S5A, B and Figs. S6A, B), suggesting that BBR reversed the oxLDL-induced blockades of autophagic flow and of LOX-1 transportation. Further, observations of the subcellular structure by transmission electron microscopy (Figs. 6B, 7B; Figs. S5C, S6C) showed the similar change of autophagosomes and autophagolysosomes in both VSMCs and VECs. In the monoculture VSMCs and VECs, there are sporadic autolysosomes and vacuoles; oxLDL exposure increased the number of typical autophagosomes. In the double-cell co-culture, autophagosomes and amphisomes/autolysosomes were seen; when oxLDL was added to the co-culture medium, autolysosome hardly present though the numbers of autophagosomes and lysosomes were notably increased, meaning a blocked autophagic flux. However, BBR addition in the co-culture plus oxLDL groups significantly increased the numbers of autolysosomes and amphisomes (Figs. 6B, 7B, Figs. S5C, S6C). In conclusion, BBR may reduce or remove intracellular waste and redundant products such as LOX-1 protein by promoting the fusion of autophagosomes to lysosomes.

### BBR induces autophagic degradation of LOX-1 and suppresses EMT in zebrafish

To verify the above results *in vivo*, we examined whether endogenous LOX-1 proteins could be trapped into autophagolysosomes using an *in-situ* immunofluorescence experiment in zebrafish.

**Figure 8:**
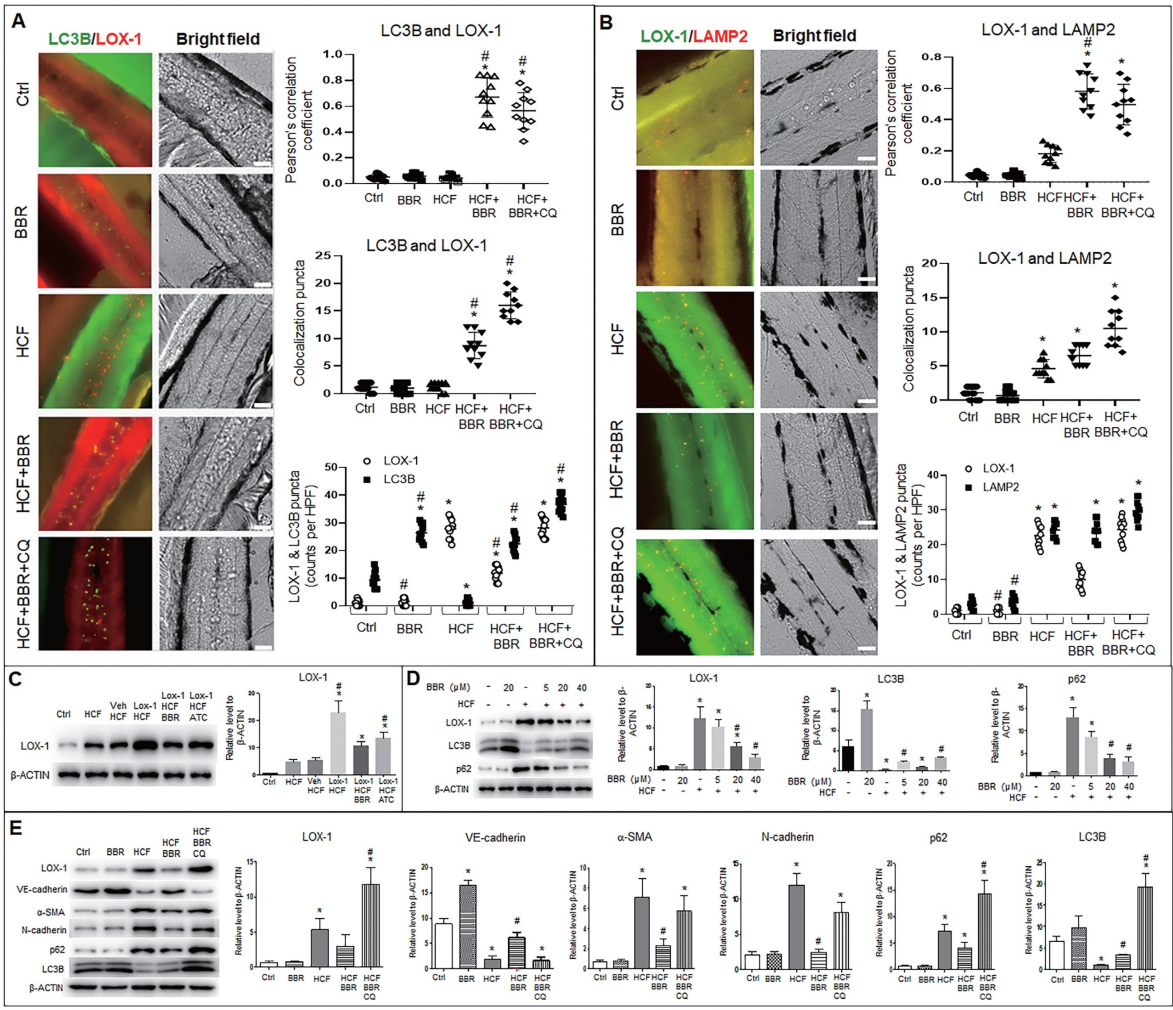
** BBR suppresses LOX-1 and EMT by activating autophagy in zebrafish.** Whole-mount immunofluorescence experiments were performed to detect co-localization between LOX-1 protein and autophagolysosomal proteins (LC3B and LAMP2). **A, B** Co-localized particles of LC3B with LOX-1 (**A**) and LAMP2 with LOX-1 (**B**) were observed in the HCF plus BBR and the HCF plus BBR plus CQ groups. The results were supported by the Pearson correlation coefficient and counts of the co-localized particles. Respective particle counts of LOX-1, LC3B and LAMP2 showed that LOX-1 protein particles was decreased by BBR treatment, but CQ suppressed the BBR effect compared to the HCF group (n=10). The bars indicate 50 μm. *P < 0.05 *vs* ctrl, #*P* < 0.05 *vs* HCF. The detailed images of LOX-1 co-localization with LC3B and with LAMP2 are shown in Fig. S7**. C** Western blot showing LOX-1 protein levels in whole zebrafish in the different treatments. **D** showing BBR effects on LOX-1 level and autophagy flux in a concentration-dependent manner. **E** Levels of EMT markers, LOX-1 and autolysosome markers in the different groups *in vivo*. The data in C-E were from 15-dpf larvae (20 zebrafish) (n = 5). *P < 0.05 *vs* ctrl, #P < 0.05 *vs* HCF.

Autophagosomes (LC3B particles) were present in the zebrafish trunk in two groups, the BBR alone and HCF plus BBR groups, and the most number was in the HCF plus BBR plus CQ group; no autophagosome was found in the HCF group, compared to the control group (Fig. 8A and Fig. S7A). However, in HCF-contained groups, LOX-1 particles notably decreased in the group of HCF plus BBR compared to the groups of HCF plus BBR with CQ; a certain number of the LC3B-LOX-1 co-localized particles were presented in the HCF plus BBR group, and the maximum number appeared in the HCF plus BBR plus CQ group, but the co-localized particles were not observed in the HCF group (Fig. 8A and Fig. S7A). Meanwhile, the combination of LOX-1 with LAMP2 was examined and showed the similar tendency as for the LC3B and LOX-1 interaction, except more lysosomes were present in the HCF group (Fig. 8B and Fig. S7B). CQ addition did not reduce the number of lysosomes but increased the number of autolysosomes. Respective counts of LOX-1, LC3B and LAMP2 particles showed that BBR decreased the number of LOX-1-positive particles, but not the number of LC3B- or LAMP2-positive particles, and CQ suppressed the BBR effects compared the HCF group (Fig. 8A, B and Fig. S7). These results suggest that HCF increased LOX-1 expression and BBR promoted LOX-1 protein degradation by promoting autophagolysosome formation *in vivo*.

Further, western blot analysis was conducted to confirm the LOX-1 protein level and autophagy flux in zebrafish. LOX-1 protein increased in the five HCF-contained groups, of which the highest LOX-1 in the HCF plus LOX-1 group; addition of BBR and Atorvastatin (ATC) decreased LOX-1 level relative to the group of HCF plus LOX-1 group (Fig. 8C). Correspondingly, the autophagy marker LC3B was upregulated, LOX-1 and P62 proteins decreased by BBR in a concentration-dependent manner (Fig. 8D), indicating that BBR promotes autophagy flux and the autophagic degradation of LOX-1 *in vivo*.

Lastly, we determined whether the LOX-1 level and EMT also could be regulated by autophagic activity *in vivo* (Fig. 8E). Compared with the control group, the quantities of endogenous LOX-1 and EMT markers (α-SMA and N-cadherin) were significantly high in the HCF group, and were reduced in the HCF plus BBR group, but increased in the HCF plus BBR plus CQ group. In contrast, VE-cadherin protein was greatly decreased in the HCF and HCF plus BBR plus CQ group, but BBR increased the VE-cadherin level in the HCF group. Moreover, the LC3B level was decreased and the p62 level was significantly increased in the HCF group, which means an impaired autophagy; when BBR was added to the HCF diet, this situation was reversed. While CQ addition elevated the levels of LC3B and p62, indicating that activity of autophagic degradation induced by BBR was abolished by CQ. These results further confirm that BBR can switch off EMT and stimulate LOX-1 degradation *via* activating *in vivo* autophagic degradation.

## Discussion

Previous studies indicated that the transmembrane glycoprotein LOX-1 serves as the oxLDL receptor that binds to and internalizes oxLDL, which is associated with endothelial dysfunction, VSMC proliferation and migration, and increased collagen synthesis and foam cell formation 31. In recent years, many clinical research and experimental studies reported that BBR could be used to treat cardiovascular diseases and hyperlipidemia by targeting several intracellular signal pathways and autophagy 17-22, 25-30. BBR could suppress expression of LOX-1 in HUVECs and monocyte/macrophages 23, 24. However, the relationship among oxLDL, LOX-1, vascular injury, autophagic activity, and BBR have not been well elucidated. In the present study, we determined that oxLDL/HCF induced LOX-1 expression in VECs and VSMCs and in zebrafish, and the LOX-1 levels are positively correlated with the “bulge-like” proliferation of VSMCs and the vascular occlusion in zebrafish. BBR exerts significant effects to decrease LOX-1 protein levels, and reverses VSMC proliferation and migration and the vascular occlusion *in vitro* and *in vivo*. These data present the integral and direct evidence that LOX-1 mediates oxLDL-induced damage of blood vessels and that BBR protects the vascular structure by promoting autophagic degradation of LOX-1 protein.

Additionally, we found that VSMCs ectopically express VEC marker VE-cadherin, and the VEC presence guides VSMC migrating to the VEC side under the condition of VEC-VSMC co-culture plus oxLDL. This phenomenon is very similar to that in the patients with hyperlipidemia-induced cardiovascular disease 32. In contrast, under cell monoculture, the VSMCs did not express VE-cadherin and did not orderly migrate to VECs, regardless of whether the cells were exposed to oxLDL. These results suggest the importance of the interaction between VECs and VSMCs in response to oxLDL stimulation.

Further, we researched the molecular mechanism underlying the effects of BBR on vascular protection. Several literatures reported BBR multiple functions, one of which is pro-autophagy. BBR can exert downregulation of mTOR protein, resulting in promotion of autophagy (since mTOR protein can increase p62, inhibit Beclin-1 and LC3B, leading to autophagy blocked) 33. In this study, BBR was demonstrated to elevate LC3B-II and to promote autophagolysosome formation in *in vitro* and *in vivo* hyperlipidemia models. BBR treatment reversed the oxLDL-induced effects, such as EMT occurrence, VE-cadherin ectopic expression in VSMC, and blocked autophagic flux with the high LOX-1 levels. Autophagy inhibitor CQ blocked the BBR actions by affecting lysosomal acidity to suppress lysosomal activity. Thus, we infer that the occurrence of EMT and VE-cadherin ectopic expression are important pathogenic factors for the changes of cell behaviors and function in vascular cells, which are mediated by LOX-1. The blocked autophagy flux caused by oxLDL inhibits the LOX-1 degradation. BBR promotes autophagosome conjunction to lysosomes, enabling transport of LOX-1 protein into autolysosomes for lysosomal degradation. Therefore, BBR's autophagy-stimulating activity maintains intracellular homeostasis of the blood vascular cells by promoting degradation of superfluous or harmful materials, such as LOX-1, in the cells.

Zebrafish serves as animal model of lower vertebrate species characterized by the following advantages benefiting the research in cardiovascular development and diseases 34. Zebrafish blood circulatory system is essentially developed at 24 h post fertilization (hpf), and is well developed at 48 hpf 35. The structure and function of their main tissues and organs are like those of mammals, and their genomic sequence has relatively high homology to that of humans 36. Studies have shown that HCF induces obesity, hyperlipidemia, and lipid deposition in blood vessel walls of zebrafish, similar to the early formation of atherosclerosis in mammalian species 37, 38. Zebrafish have been widely used as animal models of human cardiovascular diseases 39, 40. In the present study, we established a hyperlipidemia model in zebrafish induced by HCF, which simulated the typical symptoms in human, such as high cholesterol and triglyceride levels, fatty vesicle accumulation in liver. Particularly, lipid deposition on blood vessel wall caused vascular lumen blockage. Thus, the zebrafish model is thought a reliable experimental platform *in vivo* in this study. Based on this model, effects of BBR on vascular protection and lipoidemia decrease were demonstrated, which is the same as in mice.

In conclusion, this study demonstrates a link between oxLDL/LOX-1, EMT, autophagy and blood vessel integrity. Regulation of autophagy may be an effective therapeutic strategy for the oxLDL-induced cardiovascular diseases. BBR protects integrity of blood vascular system by regulating the oxLDL-LOX-1-EMT-autophagy axis. This study provides evidence showing BBR as a therapeutic candidate for treatment of hyperlipidemia-related cardiovascular diseases.

## Materials and Methods

### Animals and treatments

Zebrafish (*Danio rerio*) of the wild-type AB strain (provided by the College of Life Sciences and Technology of Tsinghua University in Beijing, China) and the transgenic Tg(fli1:GFP) strain (purchased from the National Zebrafish Resource Center, Wuhan, China) were raised under the standard laboratory conditions: a 14/10 light/dark cycle and at a temperature of 28.5±1 ºC. Fertilized eggs were maintained in an embryo medium until 5 dpf larvae. The larvae were then randomly divided into several groups and fed with different diets, i.e., 180 mg/day AP100 (Larval-AP100, Zeigler Bros, Inc., USA) plus 4% (w/w) cholesterol in the HCF group, 180 mg/day AP100 plus 4% (w/w) cholesterol and BBR (Berberine hydrochloride, purity 98%, from the National Institutes for Food and Drug Control, Beijing, China) at specified concentrations (5, 20, and 40 μM, the chosen concentrations were based on and lower than the 50% lethal dose of BBR to zebrafish in our preliminary experiments) in the HCF+BBR groups, 180 mg/day AP100 plus 4% (w/w) cholesterol and 1 μM ATC (Atorvastatin calcium, from the National Institutes for Food and Drug Control, Beijing, China) in the therapeutic medicine control group, and 30 mg/day AP100 in the control group. All the groups were fed with their specific diet for another 10 days, from 5 dpf to 15 dpf. In the cholesterol deposition experiments, 5 μg/mL of cholesterol analog (cholesteryl BODIPY 542/563 - C11; Invitrogen Co., USA) was added to the diets of the HCF and HCF plus BBR groups (20 zebrafish per group). All groups of zebrafish were fasted for 12 h before being euthanized at the end of this study phase. The experimental feeding procedure of zebrafish is outlined in Fig. S1A. The above animal studies were approved by the Institutional Animal Care and Use Committee of the Chinese Academy of Medical Sciences and conducted in accordance with the guidelines and ethics of the Chinese Council on Animal Care.

### Cell culture

A PUMC-HUVEC-T1 cell line (human umbilical vein endothelial cells; HUVECs or VEC here) was purchased from the National Experimental Cell Resource Sharing Platform of The Institute of Basic Medicine, Chinese Academy of Medical Sciences (Beijing). VECs were cultured in DMEM (Gibco) medium supplemented with 10% fetal bovine serum (FBS), 40 μU/L insulin, and 1% NEAA. A human vascular smooth-muscle cell line (HVSMC or VSMC here) was obtained from Shanghai Chengyin Biotechnology Co (Shanghai, China) and cultured in DMEM medium supplemented with 10% FBS. Both cell lines were cultured in an incubator at 37 °C and 5% CO_2_.

### LOX-1 expression vector, siRNA, and microinjection

A *lox1* expression plasmid was constructed using the zebrafish *fli1* promoter sequence as a substitute of the original CMV promoter in a pIRES2-EGFP vector, whereas the human *lox-1* full coding sequence was linked at downstream of the *fli1* promoter (pFli1ep-LOX1-IRES2-EGFP), which is functional in zebrafish vascular endothelial cells. LOX-1 overexpression was performed via injection of pFLIep-LOX1-IRES-EGFP (with pFLIep-EGFP as a vehicle control) at a concentration of 60 ng/mL and at a volume of 0.5 nL per fertilized egg at zebrafish 1-4-cell developmental stages.

### Cell transfection

LOX-1 knockdown was achieved by siRNA transfection in both VECs and VSMCs, at a concentration of 100 nM for each well. LOX-1 siRNA (sc-40185), a mixture of three different *lox-1* siRNAs, was purchased from Santa Cruz Biotechnology. LOX-1 overexpression was achieved by transfection with pIRESII-LOX1-EGFP (the expression plasmid was constructed in our laboratory) at 100 ng/well to the two types of cells using Lipofectamine 2000 (Invitrogen) reagent.

### Cell co-cultivation and treatments

VECs and VSMCs were separately cultured in a Culture-Insert 2 Well in a µ-Dish 35 mm for microscopic observation or in the two parts of a co-culture dish segregated by a rubber rod in the center of the dish (Fig. 3A). The co-cultured cells were cultured for 24 h, the inserted divider rod was removed, and then the cells were treated with oxLDL at a concentration of 100 μg/mL and BBR (National Institutes for Food and Drug Control, Beijing, China) at a concentration of 1 μM based on our preliminary experiment. After 36 h, samples were collected for examination by cell immunofluorescence analysis, Co-IP analysis, and electron microscopy. OxLDL (YB-002) was purchased from the GuangZhou Yiyuan Biotechnology Co., GuangZhou, China.

### Cell migration assay

For the transwell assay, a total of 100 μL of cell suspension (1 x 10^4^) was added to the upper chambers of a Transwell culture plate (Corning, USA, 3422, 0.8 µm). To the bottom chambers, 600 μL of the medium with or without oxLDL and BBR was added. The plate was incubated at 37 °C and 5% CO_2_ for 24 h. The cells on the upper surface of the polycarbonate films were gently removed, and the cells were fixed in 4% PFA for 30 min. Then, the cells were stained with 0.1% crystal violet for 10 min, washed 3 times with PBS, and observed under a microscope (Olympus BX53).

### Whole-mount Oil Red O staining and Nile Red staining

Zebrafish larvae (n = 20) were fixed with 4% paraformaldehyde (PFA) overnight at 4 ºC. Oil Red O staining was performed as described previously 41. Nile Red staining was performed using 0.01% Nile Red dye in PBS solution for 1 h in a dark box. Next, the larvae were washed three times with PBS under dark condition. Lipid droplets in zebrafish tissues were observed and imaged using a bright-field dissecting microscope (Olympus szx10, Tokyo, Japan).

### Biochemistry analysis of lipids

Zebrafish larvae were treated following the procedure mentioned above (n = 20 per each group), washed, and placed in a 1.5-mL EP tube. The samples were mashed by an electric high-speed homogenizer and centrifuged at 10,000 rpm for 2 min. The supernatants were separately transferred to 1.5-mL centrifuge tubes for subsequent analysis. Tissue total cholesterol (TC) and triglyceride (TGs) levels were determined using the Total Cholesterol Reagent Kit and the Triglyceride Reagent Kit (Applygen Technologies Inc., Beijing, China) according to the manufacturer's specifications.

### Histological analysis

Zebrafish larvae received the diets described above (*n* = 20 per group). After fasting for 12 h, the larvae were washed and placed in a 1.5-mL EP tube. The larvae used for paraffin section were fixed in 4% PFA overnight. For the frozen sections, larvae were directly embedded in OCT (Sakura Ltd., Tokyo, Japan) and stored at -80ºC until sectioning. HE staining of paraffin sections and Oil-Red O staining of frozen sections were performed as described previously 34. Slides were examined using an Olympus BX53 microscope (Olympus, Tokyo, Japan).

### Real-time qPCR

Total RNA was isolated with TRIzol reagent (No. 15596026, Thermo Fisher, USA) and reverse-transcribed with AMV reverse transcriptase (No. M1701, Promega, USA). qRT-PCR analysis was performed using a ROCHE Light Cycler 96 system (Roche, Basel, Switzerland). Results were analyzed using the comparative ∆∆Ct method. The human *LOX-1* primer sequences used were 5′-AAGAGAGTAGCAAATTGTTCAG-3′ (forward) and 5′-TTCAGCAACTTGGCATCC-3′ (reverse). The *β-ACTIN* primer sequences were 5′-CACCATTGGCAATGAGCGGTTC-3′ (forward) and 5′-AGGTCTTTGCGGATGTCCACGT-3′ (reverse).

### Western blot

Total proteins were extracted from zebrafish larvae or cell lysates with a RIPA lysis kit (C1053; Applygen Technologies Inc., Beijing, China), separated using 12% SDS-PAGE, and transferred to a nitrocellulose membranes (T41524; PALL, Mexico), which were blocked with 5% milk in Tris-buffered saline for 1 h at room temperature and then incubated with primary antibodies anti-LOX-1 (ab60178, Abcam), anti-VE-cadherin (ab33168, Abcam), anti-N-cadherin (13A9, mAb #14215, CST), anti-α-SMA (ab7817, Abcam), anti-LC3B (M186-3, MBL), anti-p62 (PM045, MBL), anti-LAMP2 (sc-18822, Santa Cruz), or anti-β-actin (HC201-01, ZSGB-BIO Co) overnight at 4 ºC. After washing, the blots were incubated with goat anti-mouse or goat anti-rabbit immunoglobulin G (ZSGB-BIO) for 1 h and protein bands were visualized by an immobilon western chemiluminescent horseradish peroxidase substrate (Millipore, Billerica). The western blot images are representatives of 4-6 independent experiments with corresponding histograms.

### Co-immunoprecipitation

Cell lysates were prepared using a nondenaturing lysis buffer (C1050) with protease inhibitor (cocktail, 50×, P1265-1) (Applygen Technologies, Inc.), and then separately incubated overnight with either anti-p62 (PM045, MBL) or anti-LAMP2 (sc-18822, Santa Cruz) at 4 °C, followed by a 2-h incubation step with protein A/G plus agarose (Santa Cruz Biotechnology) at 4 ºC. The immunoprecipitated complexes were eluted by boiling with SDS loading buffer for 6 min. The following immunoblot analyses were performed with the same antibodies: anti-LC3B, anti-LOX-1, anti-P62 and anti-LAMP2. An input test also was performed with the four antibodies and anti-β-actin. The electrophoresis bands of the 4 or 5 independent experiments were scanned to make histograms. Representative images are shown in the corresponding figures.

### Cell immunofluorescence

VECs and VSMCs were seeded separately in Culture-Insert 2 Wells. After experimental treatment, cells were fixed with 1% PFA for 15 min at 4 ºC. The cells were treated with specified antibodies and counterstained with DAPI dye, and then observed under a DeltaVision Imaging System (API-DeltaV; GE Healthcare) 42. The following antibodies were used for subcellular localization and co-localization analyses: anti-p62 (ab56416, Abcam), anti-LC3B (PM036, MBL), anti-LAMP2 (sc-18822/sc20004, Santa Cruz), anti-LOX-1 (ab126538, Abcam), anti-VE-cadherin (ab33168, Abcam), and anti-α-SMA (ab7817/ab5694, Abcam) antibodies. The Pearson's correlation coefficients for co-localization were obtained using DeltaVision software and are presented by scatter diagrams from 10 cells per treatment. The corresponding co-localization particles were counted and presented in the scatter diagrams.

### Zebrafish BMI measurement

In this study, 10 fish were randomly selected from 100 fish per group at 15 dpf. The fish were weighed, and their body length was measured from mouth to tail end. According to the references 43, 44, the BMI is presented as the average ratio of body weight to body length squared (kg/m^2^).

### Transmission electron microscopy

Subcellular structures were examined using transmission electron microscopy (TEM). Briefly, the cells were collected and fixed in 2% glutaraldehyde for 2 h at room temperature. Electron microscopy slices and figures were prepared by Sunny Brothers Biomedical R&D Co. Ltd. (Beijing, China). The numbers of autophagosomes and autolysosomes in VSMCs and VECs were counted and are presented in histograms (n = 20).

### Whole-mount immunofluorescence staining in zebrafish larvae

Whole-mount immunofluorescence staining was conducted as previously described 45 with minor modifications. Specifically, larvae were collected and fixed overnight at 4°C in 4% paraformaldehyde (PFA). After removing the PFA, the larvae were rinsed with PBST (PBS with 0.1% Tween-20). Then, they were treated for 30 min with proteinase K (Tiangen, RT403) and blocked in solution (10% goat serum in PBST) for 2 h. The larvae were incubated overnight at 4 °C with anti-LC3B (MBL, M152-3, 1:500 dilution), anti-LAMP2 (sc-18822/sc20004, 1:500 dilution), or anti-LOX-1 (Abcam, ab126538, 1:500 dilution) followed by a 2-h incubation step at room temperature with Alexa Fluor488- and Alexa Fluor594-conjugated antibodies (ZSGB-BIO, Beijing, China, 1:1000 dilution), respectively. The photographs were taken with deconvolution imaging technology (DV; GE Healthcare, USA).

### Statistical analysis

All data were analyzed and plotted using GraphPad Prism 7.0 (GraphPad Software Inc., La Jolla, CA, USA). For multiple group comparisons, one-way analysis of variance (ANOVA) was performed, and results with *P* < 0.05 were considered as significant.

## Supplementary Material

Supplementary figures and table.

## Abbreviations

α-SMA: alpha-smooth muscle actin
BBR: berberine
CQ: chloroquine
DAPI: 4' 6-diamidino-2-phenylindole-dihydrochlorid
EMT: endothelial-to-mesenchymal transition
GFP: green fluorescent protein
HCF: high cholesterol food
H.E: hematoxylin and eosin
LAMP2: lysosomal associated membrane protein 2
LC3B: microtubule associated protein 1 light chain 3 beta (MAP1LC3B)
LOX-1: lectin-like oxidized low-density lipoprotein receptor-1
ORO: oil-red-O staining
oxLDL: oxidized low-density lipoprotein
p62: p62/sequestosome 1 (SQSTM1)
TEM: transmission electron microscopy
VEC: vascular endothelial cells
VSMC: vascular smooth muscle cells
