# Supplementary material for: Autophagic degradation of LOX-1 is involved in the maintenance of vascular integrity injured by oxLDL and protected by Berberine
Source: Int J Biol Sci. 2023 Mar 21;19(6):1813–30. doi: 10.7150/ijbs.80958 (PMC10092756; doi:10.7150/ijbs.80958)
Supplement: Supplementary file 1 — Supplementary figures and table. [file ijbsv19p1813s1.pdf]

Supplementary Materials

Table S1. Parameters of Berberine absorption *in vivo*.

Compounds	Standard curve	Range (ng/mL)	Correlation coefficient (r)	BBR concentration in larvae water (μmol/L)	Body burden (pmols/larva)
Berberine	y=0.0283x+ 0.025	4.36-870	0.9933	5	1.09
				20	1.56
				40	2.64

LC-MS/MS method

The used liquid chromatography system was a Prominence UFLCXR LC with an autosampler (Shimadzu, Tokyo, Japan), whereas the coupled mass spectrometer was an AB 6500 Q-Trap (ABSciex, Framingham, MA). Experimental protocols were adopted from previous studies (Chen *et al*., 2017).

Chen, B., Gao, Z. Q., Liu, Y., Zheng, Y. M., Han, Y., Zhang, J. P., & Hu, C. Q. (2017). Embryo and Developmental Toxicity of Cefazolin Sodium Impurities in Zebrafish. *Frontiers in Pharmacology*, *8*, 403. https://doi.org/10.3389/fphar.2017.00403

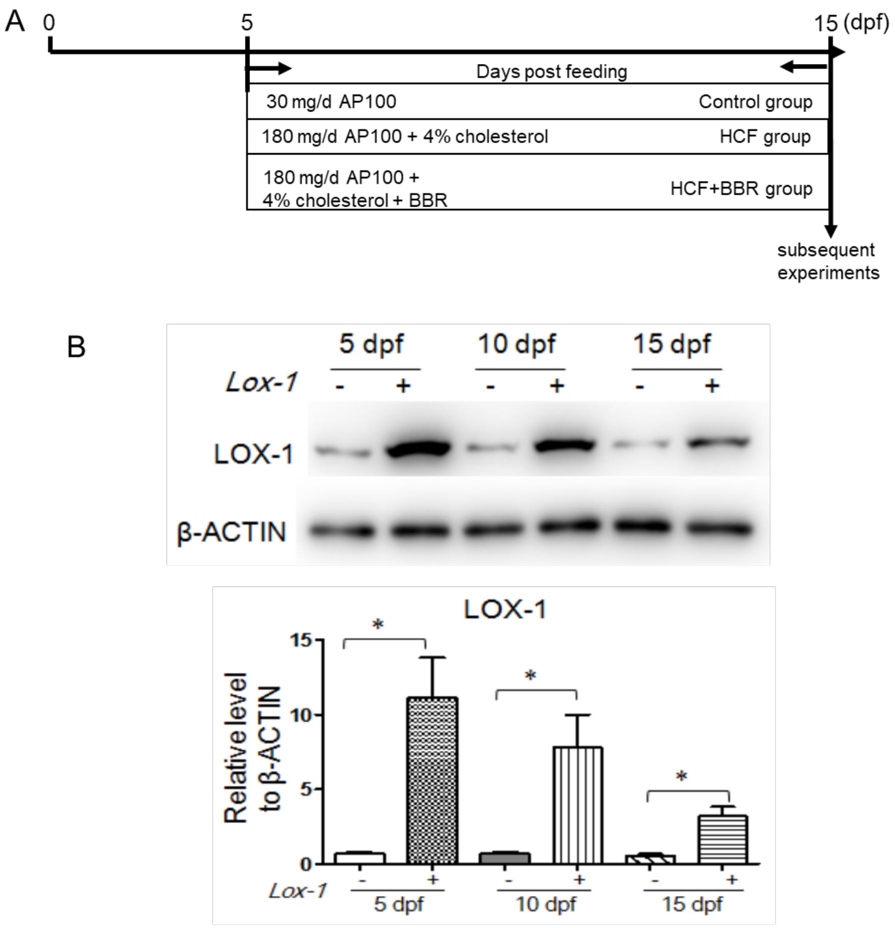

Fig. S1 The feeding flowchart and human LOX-1 overexpression in the zebrafish larvae.

A. The flowchart/timeline of the zebrafish feeding experiments. B. Western blots showing LOX-1 protein levels in the indicated developmental stages without or with human LOX-1 overexpression construct; The histogram of LOX-1 level by the band intensity scanning (n = 5). dpf: days post fertilization. *$p<0.05$.

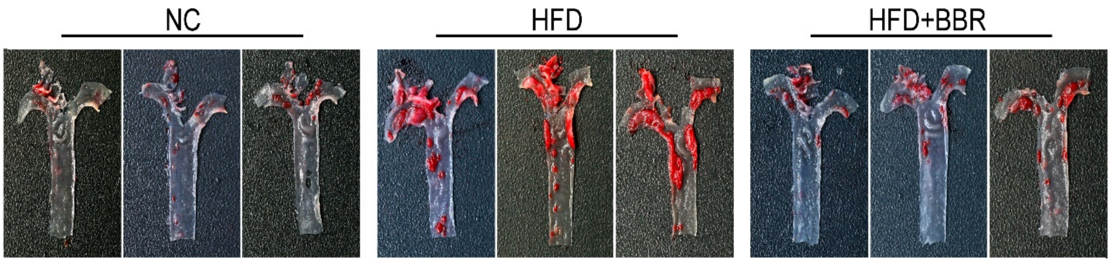

Fig. S2 BBR ameliorates arterial plaque formation in ApoE$^{(-/-)}$ mice fed a high fat diet (HFD).

HFD-fed ApoE$^{(-/-)}$ mice were treated with BBR (200 mg/kg) by gavage for 5 months. Untreated HFD-fed mice were used as model control (HFD) while ApoE$^{(-/-)}$ mice fed with standard chow diet were used as negative control (NC). At the end of experiment, the aortas from ApoE$^{(-/-)}$ mice in different group were collected. Representative images of aortic arch lesion in ApoE$^{(-/-)}$ mice using Oil red-O staining.

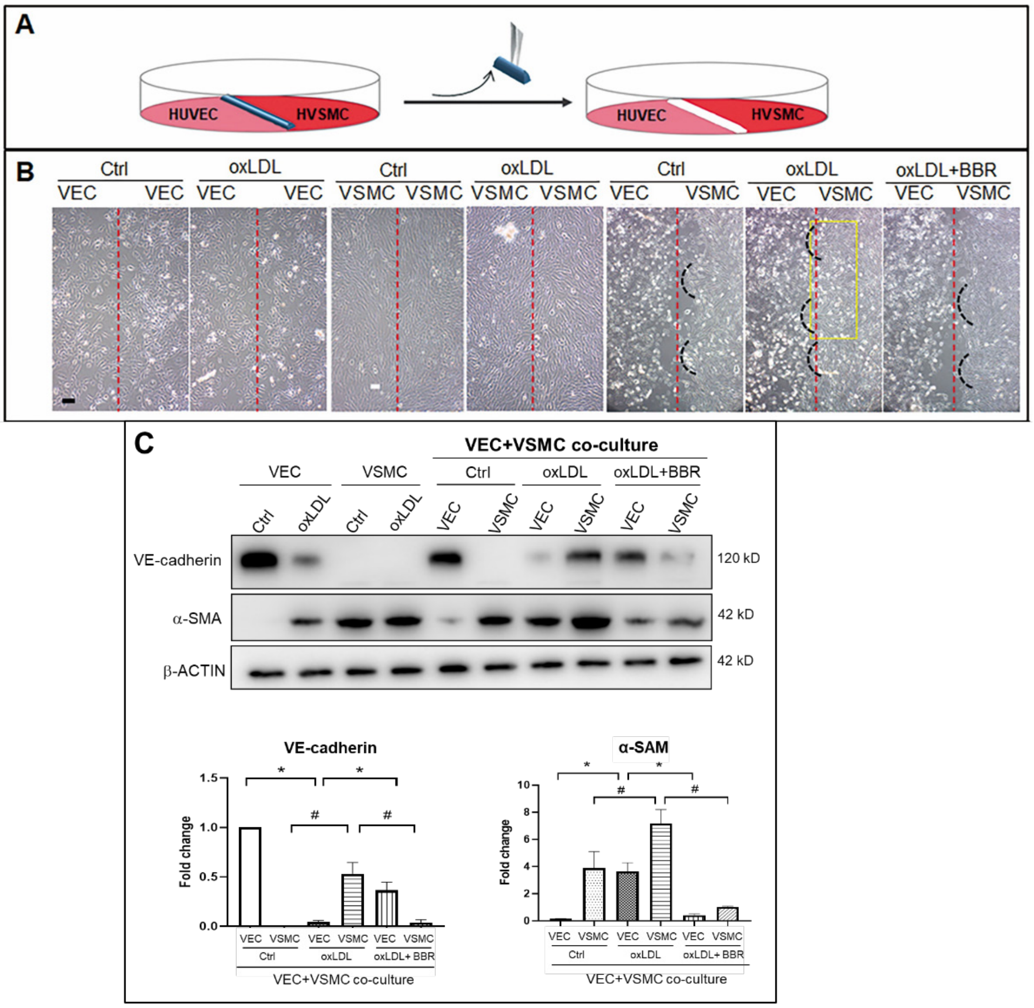

Fig. S3 VSMC proliferation and migration, and detection of the cells' marker proteins.

A. A co-cultivation device for VECs and VSMCs in this study.

B. OxLDL induced VSMC proliferation and migration with bulges toward the VEC side under VEC-VSMC co-culture. Bright-field microscopic images of BBR-related inhibition of bulging proliferation of VSMCs induced by oxLDL. The yellow open box partly indicates proliferation of VSMCs, the black dotted lines show the fronts of VSMC protuberance, and the red lines show the septal midlines between the bilateral cells. The scale bar

represents 50 μm.

C. The levels of VE-cadherin and α-SMA proteins affected by the different treatments were tested by western blot. Semi-quantitative analysis of the western blot from the co-culture condition was performed via the band intensity scanning. The values are normalized to β-ACTIN and presented as mean ± sd (n = 5). *$p<0.05$; #$p<0.05$. Semi-quantitative analysis of the western blot for Fig. 3D.

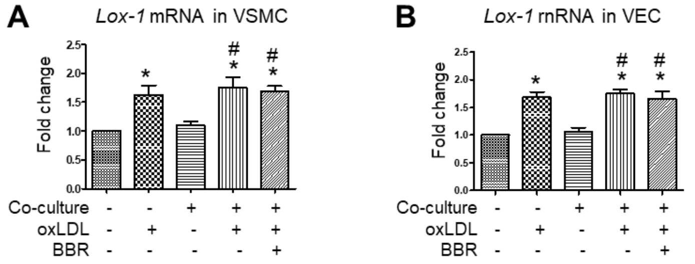

Fig. S4 Determination of *lox-1* mRNA levels by qRT-PCR.

Effects of oxLDL and BBR on the mRNA level of lox-1 in VSMC (**A**) and in VEC (**B**) under VSMC-VEC co-culture (n = 5). *$p < 0.05$ *vs* mono-culture ctrl. #$p < 0.05$ *vs* co-culture ctrl.

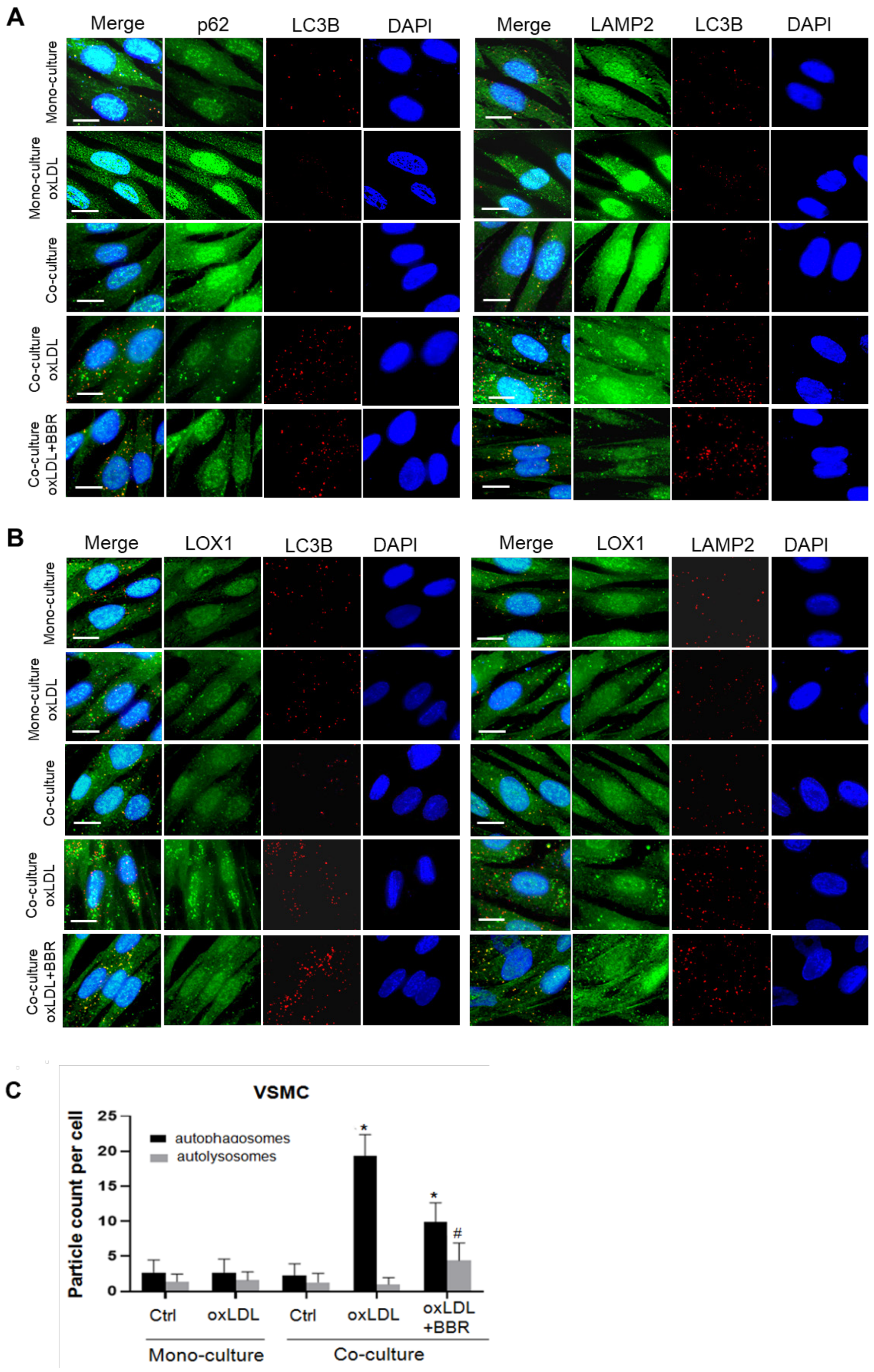

Fig. S5 Cell immunofluorescence analysis showing that Berberine promoted formation of autolysosomes containing LOX-1 protein in VSMC. (for Fig. 6)

A Under the coculture of VEC and VSMC oxLDL induced more free dots of p62, LC3B and LAMP2, but did not increase the co-localizations of P62 with LC3B and LAMP2 with LC3B; BBR addition significantly promoted their co-localization, compared with the three groups of monocultures with or without oxLDL, and co-culture without

oxLDL. **B** Similarly, under the same conditions though oxLDL induced more free dots of LOX-1, LC3B and LAMP2, but did not promote the co-localizations of LOX-1 with LC3B and with LAMP2; BBR addition prompted the colocation of LOX-1 with LC3B and LOX-1 with LAMP2. The Fig. SA and B are for Fig. 6A. **C** A histogram shows the numbers of autophagosomes and autolysosomes in the above five groups of VSMC (n = 20) (for Fig. 6B). The BBR concentration was 1 μM, and oxLDL was 100 μg/ml. The bars indicate 15 μm in (**A**) and (**B**). *$p < 0.05$ *vs* co-culture Ctrl of autophagosomes. #$p < 0.05$ *vs* co-culture Ctrl of autolysosomes.

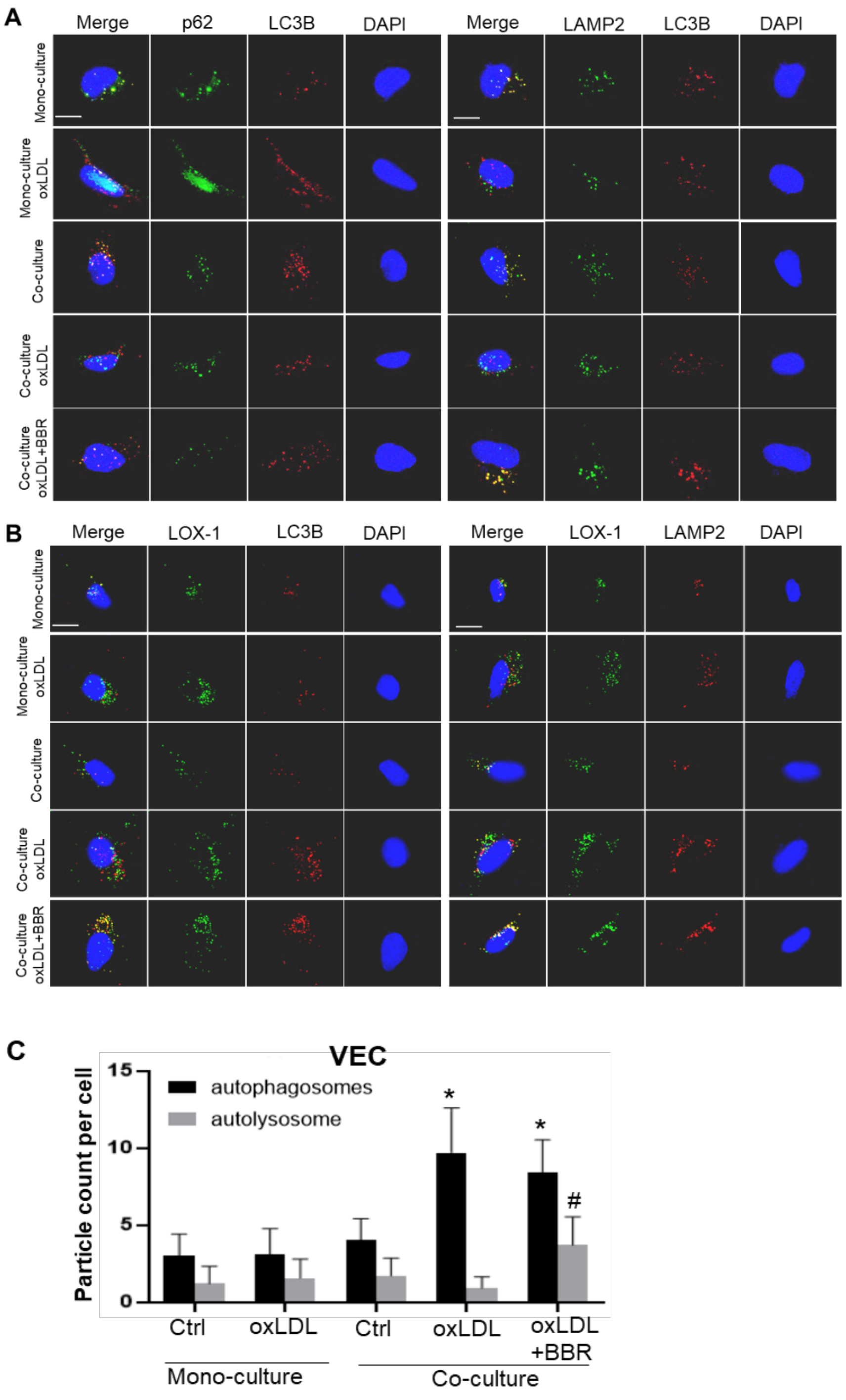

Fig. S6 Cell immunofluorescence analysis showing that Berberine promoted formation of autolysosomes containing LOX-1 protein in VEC. (for Fig. 7)

A oxLDL addition in both mono-culture and co-culture of VEC and VSMC inhibited the co-localizations of P62 with LC3B and LAMP2 with LC3B, BBR significantly promoted their co-localization, compared with the two groups without oxLDL of monoculture and co-culture. **B** Similarly, under the same conditions oxLDL inhibited and BBR increased the colocation of LOX-1 with LC3B and LOX-1 with LAMP2 in VEC. The Fig. S6A and B are for Fig. 7A. **C** A histogram shows the numbers of autophagosomes and autolysosomes in the five groups of VEC (n = 20) (for Fig. 7B). The BBR concentration was 1 μM, and oxLDL was 100 μg/mL. The bars indicate 15 μm in (**A**) and (**B**). **p* < 0.05 *vs* co-culture Ctrl of autophagosomes. #*p* < 0.05 *vs* co-culture ctrl of autolysosomes.

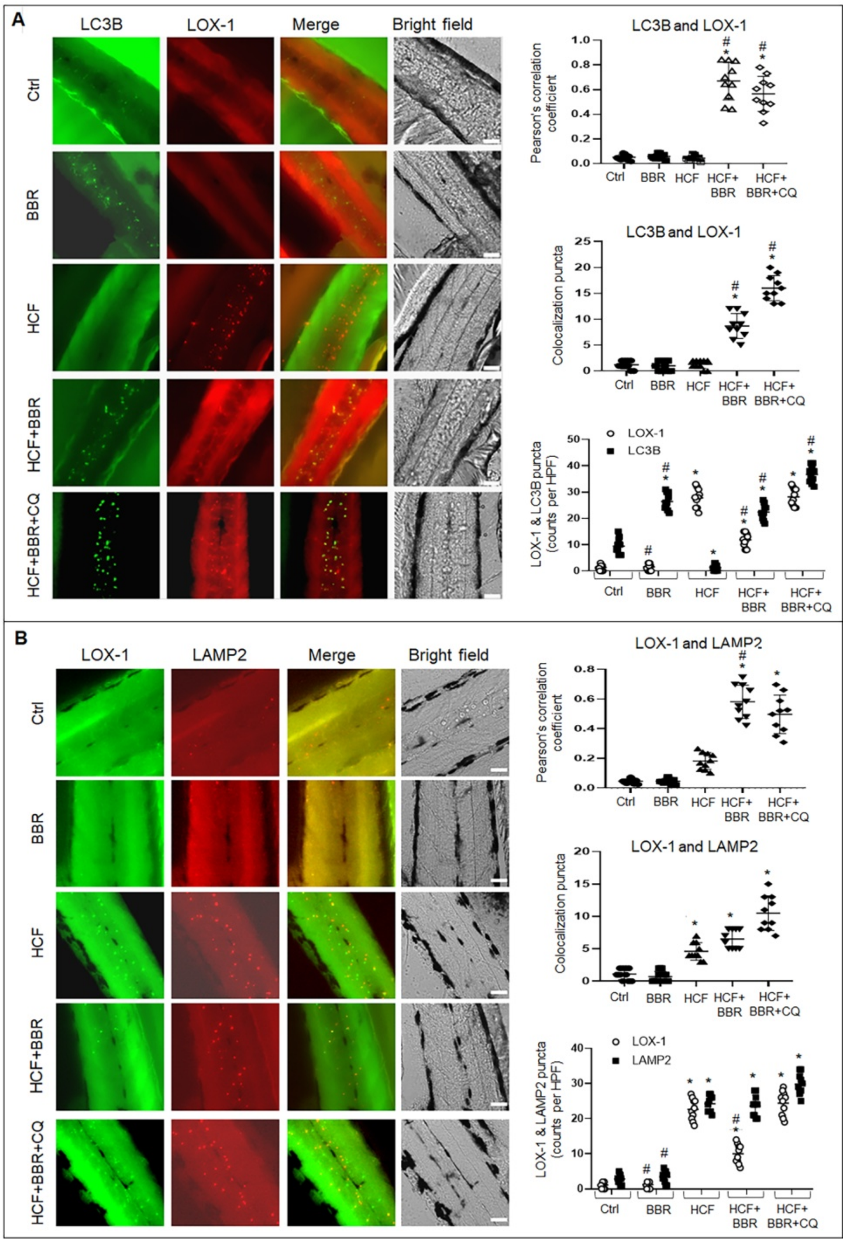

Fig. S7 BBR promotion of LOX-1 protein co-localization with LC3B and LAMP2 proteins in zebrafish. (for Fig. 8A & B)

Whole-mount immunofluorescence experiments were performed to detect co-localization between LOX-1 protein and autophagolysosomal proteins (LC3B and LAMP2). Co-localized particles of LC3B with LOX-1 (A) and LAMP2 with LOX-1 (B) were observed in both groups of HCF plus BBR and HCF plus BBR and CQ, and supported

by Pearson's Correlation Coefficient and counts of the co-localized particles. Separate particle counts of LOX-1, LC3B and LAMP2 showed LOX-1 protein decrease by BBR treatment but CQ suppressed the BBR effect compared to that in HCF group (n=10). The bars indicate 50 mm. *P < 0.05 vs ctrl: #P < 0.05 vs HCF.
